# Where is the delay? A phase-wise analysis of turnaround time in a veterinary biochemistry laboratory

**DOI:** 10.3389/fvets.2026.1846768

**Published:** 2026-09-23

**Authors:** Himalaya Bhardwaj, Sugam Singh, Rohit Kumar Jaiswal, Aseem Kumar Anshu, Ajeet Kumar, Riyaz Ahmed Siddique, Amrita Behera, Deepak Kumar, Kaushlendra Kumar, Rajesh Kumar, Pankaj Kumar, Vivek Kumar Singh, Pallav Shekhar

**Affiliations:** 1 Department of Veterinary Biochemistry, Bihar Veterinary College, Bihar Animal Sciences University, Patna, Bihar, India; 2 Livestock Product Technology, Bihar Veterinary College, Bihar Animal Sciences University, Patna, Bihar, India; 3 Department of Veterinary Pathology, Bihar Veterinary College, Bihar Animal Sciences University, Patna, Bihar, India; 4 Department of Animal Nutrition, Bihar Veterinary College, Bihar Animal Sciences University, Patna, Bihar, India; 5 Department of Veterinary Surgery and Radiology, Bihar Veterinary College, Bihar Animal Sciences University, Patna, Bihar, India; 6 Department of Veterinary Microbiology, Bihar Veterinary College, Bihar Animal Sciences University, Patna, Bihar, India; 7 Department of Veterinary Medicine, Bihar Veterinary College, Bihar Animal Sciences University, Patna, Bihar, India

**Keywords:** biochemistry, laboratory efficiency, post-analytical delay, turnaround time, veterinary biochemistry laboratory, workflow optimization

## Abstract

**Introduction:**

Turnaround time (TAT) is an important indicator of laboratory efficiency; however, limited information is available regarding its assessment in veterinary diagnostic laboratories.

**Methods:**

A retrospective and prospective observational study was conducted between April and May 2024. TAT was defined as the interval from blood sample collection to final report dissemination and was subdivided into pre-analytical, analytical, and post-analytical phases. Only complete serum biochemical profiles comprising liver and kidney function tests were included. Comparative analysis among routine weekday, weekend, and emergency workflows was performed using the Kruskal–Wallis test followed by Dunn’s post-hoc test with Bonferroni correction.

**Results and discussion:**

Among the approximately 300 biochemical samples processed during the study period, 82 routine samples fulfilled the predefined inclusion criteria and were included in the comparative analysis, comprising 54 weekday and 28 weekend samples. In addition, all eligible emergency samples were evaluated as a separate operational category. Mean total TAT was highest during weekend operations (2959.9 ± 50.3 min), followed by routine weekdays (1528.5 ± 20.0 min), whereas emergency samples exhibited the shortest TAT (54.7 ± 9.1 min). The post-analytical phase contributed the largest proportion of total TAT and demonstrated significant variation among groups (*p* < 0.001), while analytical performance remained consistent across operational categories (*p* > 0.05). Pre- and post-analytical processes accounted for 99.45 and 99.72% of total TAT during routine weekday and weekend operations, respectively. The findings demonstrate that TAT variability in veterinary biochemistry laboratories is predominantly driven by post-analytical workflow factors. Targeted improvements in reporting and validation processes may substantially enhance laboratory efficiency and diagnostic service delivery in veterinary healthcare settings.

## 1 Introduction

Turnaround Time (TAT) is a universally recognized key performance indicator in human and veterinary laboratories, reflecting the efficiency and quality of diagnostic services. TAT is defined as the total time interval from test ordering or sample collection or receipt to the delivery of validated results to clinicians, encompassing three critical phases: pre-analytical (sample collection, handling, and transport), analytical (testing procedures), and post-analytical (validation and reporting of results) (1, 2). Efficient management of TAT is essential for improving clinician satisfaction, expediting diagnosis, optimizing therapeutic decision-making, and enhancing overall patient care outcomes. Although substantial progress has been achieved in human clinical laboratories through automation, workflow optimization, and quality assurance systems (1, 3–6), delays in the pre-analytical and post-analytical phases continue to significantly influence the total TAT.

However, veterinary biochemistry laboratories, particularly in tertiary care teaching institutions, present unique operational challenges that differentiate them from human diagnostic settings. These include heterogeneous sample types from multiple animal species, variability in sample collection practices, logistical difficulties in animal handling, fluctuating sample volumes, and limitations in infrastructure and manpower (7). Such factors often contribute to inefficiencies and prolonged TAT, especially in high-throughput academic environments. In India, there is a notable lack of systematic studies evaluating TAT in veterinary laboratories. Additionally, temporal variations in laboratory performance, such as differences between morning and afternoon workloads and weekday versus weekend operations, remain insufficiently explored.

In this context, the present study sought to comprehensively evaluate turnaround time (TAT) in a veterinary biochemistry laboratory of a tertiary care institution in Patna, Bihar, by analyzing overall TAT and its phase-wise components (pre-analytical, analytical, and post-analytical) and examining temporal variations across different operational conditions, including routine weekday, weekend, and emergency workflows. Furthermore, the study aimed to identify workflow bottlenecks contributing to prolonged TAT and to highlight potential process-improvement strategies for enhancing laboratory efficiency and diagnostic service delivery in veterinary healthcare settings.

## 2 Materials and methods

This prospective and retrospective observational study was conducted at the Department of Veterinary Biochemistry, Bihar Veterinary College, Patna, India, to evaluate laboratory turnaround time (TAT) and associated workflow dynamics. Laboratory records from outpatient were collected prospectively over 2-month period and emergency veterinary cases processed between April and May 2024 were retrospectively analyzed. In this particular study, TAT was defined as the time interval from blood sample collection at the clinical unit to final dissemination of the validated laboratory report. For detailed evaluation, the total turnaround time (TAT) was divided into three standard phases: pre-analytical, analytical, and post-analytical (Figure 1). The pre-analytical phase comprised all procedures from the initiation of blood sample collection to the commencement of biochemical analysis. This phase included blood sample collection from the animal (sampling time), transportation of the sample from the clinical unit to the laboratory, sample registration and sorting, centrifugation for serum separation, and manual sorting and preparation of serum samples before loading onto the analyzer. The analytical phase commenced with loading the prepared serum sample along with the test slides onto the automated biochemistry analyzer and included biochemical testing, result generation, and completion of result entry into the laboratory record. The post-analytical phase began immediately after completion of analysis and consisted of result verification, report validation by laboratory personnel, and final dissemination of the validated signed laboratory report to the attending clinician. The duration of each phase was determined using predefined workflow time points, including sampling start time, sampling completion time, sample sorting time, manual sorting time, analyzer scan time, result entry time, and final report release time, as illustrated in Figure 1. To ensure uniformity in the evaluation of TAT, sample inclusion was strictly limited to specimens that underwent a complete serum biochemical profile, comprising both liver function tests (LFT) and kidney function tests (KFT). All biochemical analyses were performed using a fully automated, veterinary-specific analyzer (Fujifilm Dri-Chem NX600V).

**Figure 1:**
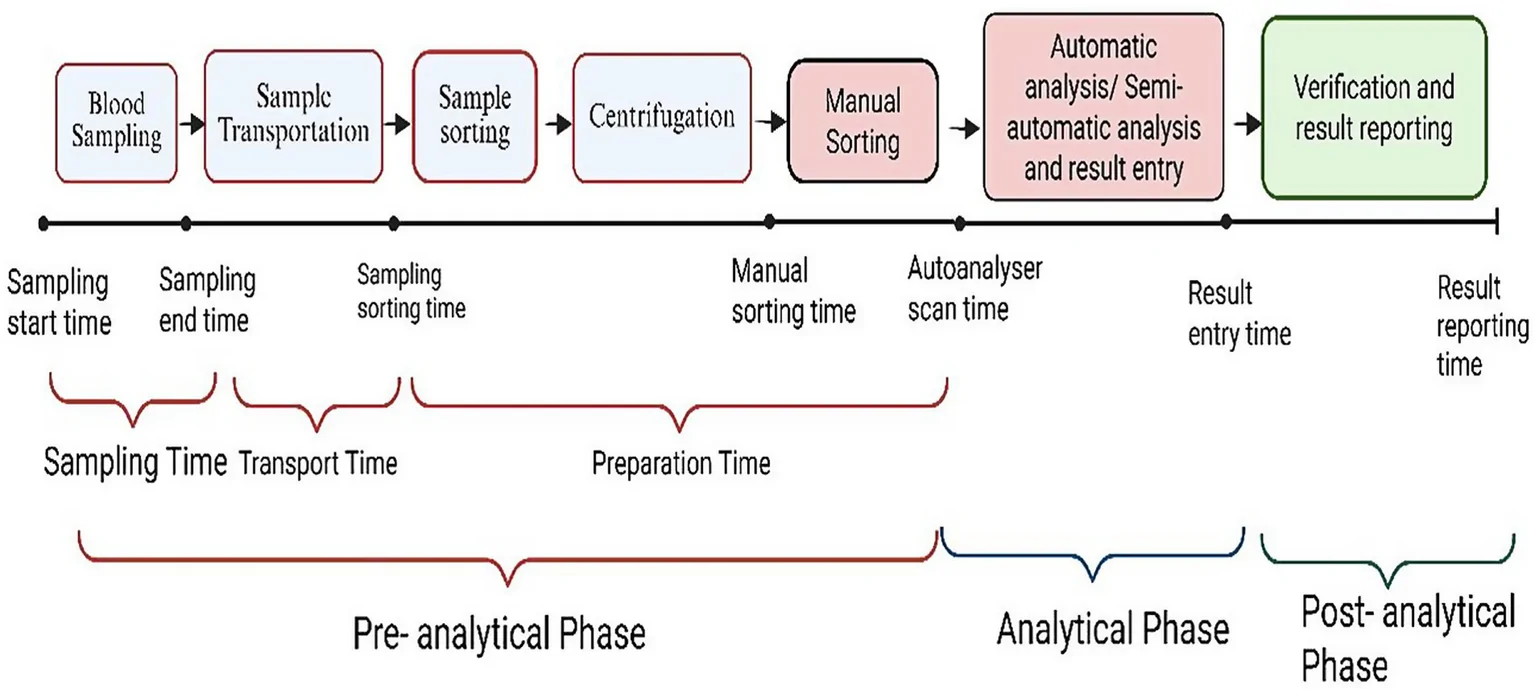
Workflow diagram illustrating the pre-analytical, analytical, and post-analytical phases used for turnaround time (TAT) measurement in the veterinary biochemistry laboratory. The workflow was established based on the routine operational practices of the laboratory and represents the sequential progression of samples from blood collection through transportation, sample sorting, centrifugation, manual sample preparation, automated biochemical analysis, result verification, validation, and final report dissemination. The predefined time points were used to classify TAT into pre-analytical, analytical, and post-analytical phases. This workflow served as the basis for identifying operational bottlenecks and evaluating phase-wise contributions to the overall TAT under routine weekday, weekend, and emergency laboratory conditions.

The study was conducted over a 61-day period from 1 April to 31 May 2024. Of these, 44 routine weekdays (Monday–Friday) and eight Saturdays (weekend category) were included in the comparative TAT analysis. One Saturday was excluded because no complete serum biochemical profile was available for evaluation. In addition, all emergency samples fulfilling the study inclusion criteria and processed during the study period were analyzed separately owing to their prioritized handling and reporting procedures. For operational comparison, the weekend category comprised samples processed on Saturdays. Because Sunday was a non-operational day, regular report validation and dissemination were often carried out on Monday, the following working day, even though analytical testing was finished on the same weekend day of sample receipt. During the study period, approximately 300 samples were received for biochemical analysis. To ensure methodological consistency, only samples comprising a complete serum biochemical profile, including both liver and kidney function tests, were eligible for inclusion. Based on these criteria, 82 samples were included in the final analysis, comprising 54 samples processed during routine weekdays and 28 samples processed during weekend operations.

To minimize selection bias, all consecutive serum biochemical profiles meeting the predefined inclusion criteria and processed during the study period were included in the analysis. Turnaround time measurements were obtained from laboratory records prospectively and analyzer-generated timestamps, thereby reducing the potential for measurement-related observer bias. Workflow observations were performed using standardized procedures to ensure consistency in the operational flow. Descriptive statistics were calculated for all turnaround time (TAT) phases and expressed as mean ± standard error (SE). As the data did not satisfy the assumptions of normality and homogeneity required for parametric analysis, comparisons among routine weekday, weekend, and emergency groups were performed using the non-parametric Kruskal–Wallis H test. When significant differences were detected, pairwise comparisons were conducted using Dunn’s post-hoc test with Bonferroni correction to account for multiple testing. Statistical significance was established at *p* < 0.05.

## 3 Results

A comparative analysis of the turnaround time (TAT) under different operational conditions demonstrated significant variations in the overall laboratory performance. The mean total TAT was highest in weekend samples (2959.9 ± 50.3 min), followed by routine weekday samples (1528.5 ± 20.0 min), while emergency samples exhibited the lowest TAT (54.7 ± 9.1 min) (Table 1).

**Table 1:** Comparative analysis of TAT across different conditions.

Parameter	Routine Weekday	Routine Weekend	Emergency Cases	*p*-value (Kruskal-Wallis)
Pre-analytical (min)	225.6 ± 1.2	225.1 ± 1.2	30.0 ± 6.2	*p* < 0.001
Analytical (min)	8.4 ± 0.9	8.4 ± 1.1	6.7 ± 3.1	NS (*p* = 0.691)
Post-analytical (min)	1294.5 ± 20.3	2726.2 ± 50.5	16.7 ± 5.3	*p* < 0.001
Total TAT (min)	1528.5 ± 20.0	2959.8 ± 50.3	54.7 ± 9.1	*p* < 0.001

In this study, phase-wise evaluation demonstrated that the post-analytical phase contributed the largest proportion of total turnaround time (TAT) and exhibited significant variation among groups (*p* < 0.001). Similarly, pre-analytical times differed significantly across operational categories (p < 0.001), primarily due to the substantially shorter processing times observed in emergency cases. In contrast, no statistically significant difference was observed in the analytical phase (*p* = 0.691), indicating consistent analytical performance irrespective of workflow conditions (Table 1). Furthermore, proportional analysis revealed that the combined pre-analytical and post-analytical phases accounted for the overwhelming majority of the total TAT in this tertiary veterinary biochemistry laboratory, contributing 99.45% on routine weekdays and 99.72% on weekends, whereas the analytical phase contributed less than 1% under these conditions. In emergency cases, the relative contribution of the analytical phase increased to 12.57%, whereas the combined pre- and post-analytical phases accounted for 87.43% of the total TAT (Table 2). This shift reflects a substantial reduction in pre- and post-analytical delays under prioritized workflow conditions and indicates that total TAT in routine laboratory settings is predominantly driven by non-analytical processes, with the analytical phase becoming proportionally more prominent only when systemic inefficiencies are minimized. Kruskal–Wallis analysis revealed a highly significant difference in total TAT among the three operational categories (*p* < 0.001). Subsequent Dunn’s post-hoc analysis with Bonferroni correction confirmed significant pairwise differences between routine weekday, weekend, and emergency groups (*p* < 0.05), highlighting the influence of operational workflow and reporting practices on laboratory efficiency

**Table 2:** Percentage contribution of TAT Phases to total TAT.

Phase	Routine weekday (%)	Routine weekend (%)	Emergency cases (%)
Pre-analytical	14.76%	7.61%	56.15%
Analytical	0.55%	0.28%	12.57%
Post-analytical	84.69%	92.11%	31.28%
Total	100.00	100.00	100.00

## 4 Discussion

The present study provides a comprehensive evaluation of Turnaround Time (TAT) in a veterinary biochemistry laboratory and demonstrates that laboratory efficiency is significantly influenced by operational conditions and workflow prioritization (1, 8–10). A key finding of this study was the substantial variation in total TAT across different categories, with emergency samples exhibiting markedly reduced processing times compared to routine weekday and weekend samples. This pronounced difference underscores the critical importance of rapid diagnostic reporting in veterinary emergency settings, where timely intervention is essential for improving clinical outcomes in veterinary patients (11). The ability of the veterinary diagnostic laboratory to achieve extremely low TAT in emergency situations highlights the inherent efficiency of the existing analytical infrastructure when supported by prioritization mechanisms (12, 13).

The study’s primary observation was that variability in Turnaround Time (TAT) was mainly influenced by the post-analytical phase. While pre-analytical times varied significantly across operational categories, primarily due to the expedited processing of emergency samples, analytical performance remained consistent, with no significant differences among groups. This consistency suggests that the biochemical testing process was standardized and well-controlled. The notable differences in total TAT, despite stable analytical performance, highlight that delays were chiefly due to workflow-dependent factors outside the analytical phase (2, 5). Importantly, the pre-analytical phase consistently required more time than the analytical phase across all categories, reflecting the procedural demands of sample collection, transportation, registration, handling, and preparation prior to testing.

In contrast, the post-analytical phase emerged as the principal bottleneck, contributing disproportionately to the total TAT across all non-emergency conditions. The marked prolongation of post-analytical time during weekend operations highlights the influence of workflow-related constraints, including delayed report dissemination, limited staffing, and the absence of continuous automated reporting systems. In the present study, routine samples processed on Saturdays were often validated and released on the following working day because Sunday was a non-operational day, thereby substantially extending the post-analytical interval. Similar observations have been reported in human laboratory settings, where post-analytical delays are frequently attributed to inefficiencies in result verification, communication, and reporting processes (3, 14). However, the magnitude of delay observed in the present veterinary setting was considerably greater, suggesting that veterinary laboratories, particularly those within tertiary care institutions, may face more pronounced systemic challenges related to resource constraints, workflow organization, and limited integration of laboratory information management systems.

The significant increase in TAT during weekend operations further reinforces the role of organizational factors in determining the efficiency of the laboratory. The near doubling of TAT on weekends compared to weekdays reflects reduced workforce availability, interruptions in workflow continuity. These findings are consistent with previous reports in human healthcare systems, where weekend effects have been associated with reduced service efficiency and delayed clinical decision making (14, 15). In veterinary practice, where timely diagnosis directly impacts both individual animal care and broader herd health management, such delays may have significant consequences (15).

Emergency samples demonstrated substantially reduced pre-analytical and post-analytical delays owing to prioritized handling and expedited reporting procedures (13, 16). While these findings illustrate the impact of workflow prioritization on reducing TAT, emergency samples constituted a relatively small proportion of the overall workload and therefore should not be interpreted as a directly achievable benchmark for routine laboratory operations. Nevertheless, the marked reduction in TAT observed under prioritized conditions suggests that a considerable proportion of routine delays arise from modifiable workflow and reporting practices rather than analytical limitations.

Overall, the findings of this study indicate that TAT variability is primarily influenced by workflow design and staffing patterns, rather than analytical performance. The identification of the post-analytical phase as the principal contributor to prolonged TAT highlights an important opportunity for quality improvement in veterinary diagnostic laboratories. Targeted interventions, including automated result validation, strengthened laboratory information management systems, real-time digital report dissemination, streamlined reporting pathways, and optimized staffing strategies, may substantially reduce post-analytical delays and improve overall laboratory efficiency. Adoption of selected process optimization approaches that have been successfully implemented in advanced human laboratory systems could further enhance diagnostic service delivery and support timely clinical decision-making in veterinary healthcare settings (17).

In conclusion, this study provides a comprehensive phase-wise evaluation of turnaround time (TAT) in a veterinary biochemistry laboratory and identifies the post-analytical phase as the principal contributor to prolonged TAT. Routine weekend operations exhibited substantially greater TAT than routine weekdays, highlighting the scope for greater automation and streamlining of post-analytical processes within the existing laboratory workflow. The findings demonstrate that overall laboratory efficiency may be further enhanced through optimization and automation of post-analytical processes, rather than through changes in analytical performance. To the best of our knowledge, studies providing a detailed phase-wise assessment of TAT and its operational determinants in veterinary biochemistry laboratories remain limited, particularly in tertiary veterinary teaching institutions. By identifying critical workflow bottlenecks and highlighting the disproportionate contribution of post-analytical delays, the present study provides practical evidence to guide quality-improvement initiatives aimed at strengthening reporting systems, optimizing workflow management, and enhancing diagnostic service delivery in veterinary healthcare settings. These findings may serve as a foundation for future multicentric studies and the development of standardized TAT benchmarks for veterinary diagnostic laboratories.

## Data Availability

The raw data supporting the conclusions of this article will be made available by the authors, without undue reservation.
